# Letter from Vienna—Clinics for Skin Diseases and Syphilis,—Billroth’s Operation, &c.

**Published:** 1879-07

**Authors:** 


### Miscellaneous.

### Letter from Vienna.

*Clinics for Skin Diseases and Syphilis.—Billroth's Operation.—Clinical Cases, &c.*

MR. EDITOR:—Vienna continues to be very attractive to students of medicine. Nearly all nations are represented here this winter, including a large number of English-speaking people, one hundred and eight of whom are Americans. The abundant material to which the student has full access, the large number and the excellence of the private courses, and the fact that they are so arranged that one can go from course to course all day long without losing time, are the wide-known attractions of the Vienna system.

Skin is decidedly the most fashionable study. In this branch of medicine there are six clinics daily. Professors Hebra, Neumann, and Kaposi are the best, the two last being the most popular among the Americans. Professor Kaposi has just come out with a new book, which is essentially the same as his lectures, and is remarkably clear and explicit, especially in regard to treatment. In the Hebra clinic one is struck with the rapidity and accuracy in diagnosis, and the simplicity and efficacy of the treatment. Hebra's spoon scrapers are extensively used in the treatment of epithelial cancers, lupus, moles, etc. With these instruments it is almost impossible to scrape away anything but the diseased tissue. Quite large epithelial cancers of the face are at first spooned out as much as possible, and later the returning disease is continually cauterized with nitrate of silver until the granulations are healthy. The resulting scars are often better than could have been expected from a plastic operation. Lupus is usually treated in much the same way, but when the disease is extensive or the patient especially sensitive, a strong pyrogallic-acid ointment is applied. At the end of two or three days the diseased parts are found to be necrosed, and soon slough out, leaving healthy skin on the sides and granulations beneath. The experiments with chrysarobin and pyrogallic acid in the treatment of psoriasis, herpes tonsurans, etc., are still continued, and have already been mentioned in your pages by Dr. Garland.

Syphilis seems to be well understood, and is excellently taught. The wards of Professor Sigmund are noticeable for the order,—the discipline of the nurses, who do all the dressings in both male and female wards,—and for the scrupulous personal cleanliness of the patients. Professor Sigmund himself has given only four lectures this winter. His assistant, Dr. Mrazk, gives a very good private course, where one can get a large experience in diagnosis and treatment in a short time. The well-known inunction cure is extensively used ; also the subcutaneous injection of either peptone quicksilver or the albuminate of quicksilver.

Professor Sigmund claims that wounds heal as well in syphilitic subjects as in non-syphilitic; accordingly, if a chancre is so situated under the prepuce that it cannot be taken care of, the patient is circumcised, and not only the prepuce but as much of the induration as is inconsistent with the symmetry of the organ is also removed. Circumcision is almost always done if the chancre is so situated that it can be removed with the prepuce. When the wound unites by first intention, which often happens, there is a clear gain of two months' treatment of the chancre. These wounds usually do very well, except in cases where the chancre is gangrenous or diphtheritic.

Indolent suppurating buboes, syphlitic or otherwise, are not simply laid open, but all the tissue of the upper part is cut away down to the edge of the cavity, leaving a flat, open wound, and if the pus has burrowed under the glands they too are excised.

In surgery Professor Billroth attracts a large class, a few of whom are Americans. He usually has something interesting. A few days ago he removed a larynx, and early in the winter he exhibited a man with a gastric fistula. We were somewhat surprised at the treatment of a hip dislocated on the dorsum ilii of four months' duration. He made no attempt to reduce it by manipulation on account of its long standing, but opened the joint by a long incision from the buttock down on to the back of the thigh; he then "cut all the muscles that prevented its reduction," and with the aid of several assistants brought the leg into position; then putting his hand into the wound brought out the head of the bone, which had been broken off by the reduction. His wards are remarkably clean and orderly. He usually operates under carbolic spray, and applies a very large dressing consisting of simple disinfected gauze containing no antiseptic whatever, over which is a large quantity salicylic oakum or cotton, and outside of all a starch bandage, to render the part immovable and the dressing secure. The solutions for washing the wound, etc., are thymol. Heavy flaps in wounds are held in place by deep wires secured by lead balls.

In diseases of the urinary organs Dr. Ultzmann gives a good course. He has made a special study of the pathology and treatment of spermatorrhœa. Not to mention many smaller points, he says that in these cases the pars membranacea and prostatica is especially sensitive to the sound, and that the mucus membrane in that region is excessively hyperæmic as seen with the endoscope. Towards such a condition of the urethra and a relaxation of the ducts of the vesiculæ seminales he aims his local treatment, which consists for the most part in the application of a sound cooled by a double current of water and astringent suppositories introduced by Dittel's *porte-remède.* His results are often good. He points out the danger and demonstrates cases of male sterility after gonorrhœa caused by inflammatory obliteration of the ejaculatory ducts. He insists on the risk of entirely emptying a paralytic bladder at the first catheterization, on account of the hyperæmia *ex vacuo* which will follow in proportion to the thickness of the wall and to the amount of urine that constantly remained in the bladder. The immediate danger is a hæmorrhage into the kidneys or bladder. The worst result to be thought of is a fatal nephritis; the least is a more or less severe cystitis. His procedure is partly to empty the bladder at first, and to substitute a carbolic solution (one half per cent.) for the urine, gradually diminishing the amount until the bladder is empty, at the same time applying massage and electricity to the bladder region. He has just written a book on the examination of urine, which contains references to his well-known atlas of urinary sediments. This book explains many new points, and is of especial value to his pupils.

Midwifery is popular, because the students are allowed to examine all the cases and to perform the smaller operations. The courses were all stopped for a time on account of the large number of puerperal fever. The instructors make a great point of external examination and diagnosis. Last May Dr. Schanta, Professor Späth's assistant, having read of some cases of abortion accidentally caused by pilocarpine, resolved to test its power in that direction. Accordingly, he caused a woman to abort by subcutaneous injections of pilocarpine, and published the case. Since that time he has continued to experiment with the drug. Of late he has used it with good results in those cases in which ergot is contraindicated from the fact that the os is not fully dilated. These experiments will soon be published.

It has long been the custom here to cut and tie the cord as soon as it is pulseless. Within a few months a set of experiments have been instituted in Professor Späth's ward which show that the child, the cord not being cut, gains on an average about seventy grammes in weight between the time it is born and the time when the placenta comes away, and that a large proportion of this gain takes place after the cord has stopped beating. In the light of these experiments the cord is not tied until the placenta has come away, in order that the child may get the benefit of as much blood as possible.

In clinical medicine Professor Bamberger has of late become very popular, and he certainly impresses one as a man of great learning and ability. In the most difficult cases he runs rapidly through the symptoms, pathology, and treatment, discussing the latest theories in point, and giving the possibilities, probabilities, and certainties in regard to the diagnosis in a mechanically logical way seldom heard in medicine. The autopsies show him to be remarkably accurate. There is a good private course in his wards under his assistant, where students are allowed to examine the cases, and oftentimes the case to be shown by Bamberger in the next morning's clinic. In this department the instructors seem to be especially advanced in the interpretation of the auscultation of the heart, Professor Bamberger having written an excellent book on the subject some years ago. Here we have met a disease new to us, and said to be recognized only in Vienna, namely, workers in mother-of-pearl often appear here with periostitis or valvular disease of the heart, or both together. At the autopsies of these cases pearl powder is found deposited on the periosteum and bone; also on the affected valves of the heart.

Dr. Oster gives a course on the stomach and intestines. Here we have seen startling results from the mechanical washing out of the stomach and the irrigation of the intestines. It is astonishing to see with how little effort or discomfort the patients without assistance swallow the rubber tube through which the water is introduced.

Dr. Monti gives a very popular course on children. In pathological anatomy Dr. Chiari, Professor Heschl's assistant, gives a very good course. Curious and rare specimens are constantly appearing out of the enormous mass daily exhibited. The results of operations are often instructive. Early in the winter, on exploring a wound of the neck, from which some glands had been removed, the carotid artery was found to have been nicked, the jugular vein to have been cut and tied, and the phrenic nerve tied. It is an interesting fact that this patient had experienced no special difficulty in breathing since the operation. Within a few weeks three cases have appeared, the results of extirpations of the uterus; in each case a ureter was cut, and in two of the cases the ureters were cut and tied.

Dr. Chiari has of late examined eight hundred rectums with reference to the pathology of anal fistulæ. He has noticed that Morgagni's glands, situated just inside the anus, where the mucous membrane begins to take on the characteristics of true skin, although very shallow in young individuals, are often quite deep in old people, and that this appearance is often increased by the columnæ Morgagnii, the parts between the glands being thickened by hæmorrhoidal veins. He has succeeded in obtaining a series of specimens which show these glands in a great variety of conditions and sizes,—some shallow, some quite deep, some very deep and extending through the sphincter ani. On the other hand, he has found mucous membrane lining parts of the tracts of complete fistulæ. His idea is that when the rectum is full of fæces and the sphincter contracts, these glands, as parts of less resistance, are slowly pushed out, dragging the mucous membrane behind; that once having pushed through the sphincter ani so that the fæcal matter cannot return to the bowel, the stagnating masses excite ulceration, and come gradually, though not always, directly to the surface. He thinks that the fact that the surgeon always looks for the opening of the fistula just inside the anus is a support to his theory. Whether this theory be accepted or not, perhaps the facts that some fistulous tracts are lined in part by mucous membrane will explain their obstinacy in healing, and suggest the inadequacy of treatment by simply cutting the outer wall. J. W. E.
*Boston Medical and Surgical Journal.*

